# Congenital Stationary Night Blindness (CSNB)—Case Reports and Review of Current Knowledge

**DOI:** 10.3390/jcm14041238

**Published:** 2025-02-13

**Authors:** Magdalena Durajczyk, Wojciech Lubiński

**Affiliations:** Second Chair, Department of Ophthalmology, Pomeranian Medical University, 70-111 Szczecin, Poland; lubinski@pro.onet.pl

**Keywords:** congenital stationary night blindness, impaired night vision, retinal electrophysiology, negative ERG

## Abstract

**Purpose:** To present the current state of knowledge and our diagnosed patients with congenital stationary night blindness. **Material and methods:** Data from the PubMed database on CSNB and the presentation of patients with complete and incomplete forms of this condition. Patients underwent routine ophthalmologic examination, optical coherence tomography, and full-field elecroretinogram (ERG-ISCEV), ON-OFF ERG. **Results:** CSNB is a group of rare, non-progressive retinal diseases characterized by impaired night vision from birth, reduced visual acuity, myopia, nystagmus, and strabismus. Color vision and fundus imaging are most often normal. CSNB is mainly inherited autosomal recessively. Eighteen genes with more than 360 pathogenic variants have been detected in this condition. The effect of gene mutations is to damage the function of rods (Riggs type) and bipolar cells of the retina (Schubert–Bornstein type). The key diagnostic test in CSNB is ERG. In diagnosed cases of complete CSNB the following types have been registered: rod ERG absent, rod–cone response negative (ON bipolar cell defect), and photopic ERG enlarged a-wave. In incomplete CSNB-rod ERG-subnormal, rod-cone response-negative (bipolar cell defect ON, OFF), photopic ERG-subnormal with a double peak in the flicker fusion frequency. Knowledge of the phenotypic changes associated with various gene pathogenic variants is still very limited, hindering the ability to correctly diagnose a patient based on clinical examination and additional ophthalmologic tests. However, some phenotypic features found in our cases were consistent with pathogenic variants previously described in the literature and helped to make a diagnosis that was proven by genetic testing. **Conclusions:** Congenital stationary night blindness should be considered in the diagnosis of retinal diseases manifesting with impaired night vision. A correct diagnosis is especially important for the patients, as it is nonprogressive, unlike other diseases that should be considered in the differential diagnoses.

## 1. Introduction

Retinal dystrophies, a type of optic nerve neuropathy, are major causes of severe, usually progressive vision loss in children [1,2]. Congenital stationary night blindness (CSNB) is a **non-progressive** retinal disease characterized by the following: impaired night vision from birth, visual acuity that may be normal or reduced to 20/200, photophobia, myopia, nystagmus, and strabismus (occurring in 50–70% of cases) [3], with preserved color vision [4,5]. CSNB is rare, but it may be underdiagnosed because clinical signs can be overlooked in young patients and there is limited availability of appropriate electrophysiological and genetic testing. In CSNB, there is a disruption of signal processing in photoreceptors, retinoid metabolism in the retinal pigment epithelium (RPE), or signal transduction by retinal bipolar cells [5]. The disease can be inherited in an X-linked (most common), autosomal recessive, or autosomal dominant manner.

The electroretinogram (ERG) is commonly used in the diagnosis of rod, rod–cone, and cone dystrophies. Clinically, the ERG is valuable for diagnosing a range of inherited and acquired retinal and genetic diseases. In congenital stationary night blindness, the ERG is crucial because the fundus is often normal. A negative ERG observed in CSNB suggests disorders in the middle retinal layer. In addition, a negative ERG is characteristic of other acquired and congenital diseases that should be considered in the differentiation of CSNB (Table 6). The aim of this article is to present the current state of knowledge and our patients diagnosed with congenital stationary night blindness.

## 2. Types of CSNB Based on Electrophysiological Findings

The negative type of electroretinogram (ERG) characteristic of CSNB was first identified by Schubert and Bornschein in 1952 [6]. In patients with a normal fundus or uncharacteristic changes (typical of axial myopia), two types of CSNB are identified:

1. The Riggs type is characterized by selective dysfunction of rod receptors, (DA—dark adaptation, LA—light adaptation):
  - DA 0.01 ERG—not detectable;
  - DA 3.0 ERG—reduction in amplitude of a- and b-waves;
  - LA 3.0 ERG—normal response [7];
2. The Schubert–Bornschein type is divided into two subtypes, with characteristic ERG results for the complete (cCSNB) and incomplete (icCSNB) forms [8] (Table 1 and Table 2).

## 3. Types of CSNB with Fundus Lesions

1. Fundus albipunctatus (FAP) is a rare inherited retinal disorder characterized by the presence of small white or yellowish-white punctate lesions in the mid-periphery of the fundus, sparing the macula at the level of the retinal pigment epithelium. Using fundus autofluorescence (FAF), the accumulation of lipofuscin in the RPE of a patient with FAP can be observed. The disease manifests in early childhood and primarily affects rod photoreceptors [9].
  In ERG examination the following is observed:
  - DA 0.01 ERG—absent, but after prolonged dark adaptation (120 min) there is full recovery of the b-wave;
  - DA 3.0 ERG—reduction in the amplitude of a- and b-waves, with b-wave reduction possibly greater than a-wave reduction (negative ERG pattern);
  - LA 30 Hz—reduction in interpeak values;
  - LA 3.0 ERG—normal.
2. Oguchi disease is a rare form of congenital stationary night blindness, characterized by the Mizuo–Nakamura phenomenon (a metallic sheen across the entire retina, which disappears after approximately 3 h of dark adaptation). The ERG is similar to the Riggs type—prolonged dark adaptation results in an improved rod response. Further research on the genes involved in phototransduction and light adaptation is needed to determine the pathogenesis of this rare disease [10].

## 4. Characteristics of CSNB Based on Inheritance Patterns, Fundus Lesions, and Presence of Nystagmus

In 2020, Faris Almutairi et al. created a classification scheme for CSNB, based on inheritance patterns, fundus lesions, and the presence of nystagmus [11]. It shows that the following is observed in **autosomal recessive inheritance of CSNB:**

1. A **normal** fundus and an **absence of nystagmus** suggest a pathogenic variant in the *GNB3* gene;
2. An **abnormal** fundus but **no nystagmus** suggests Fundus albipunctatus (pathogenic variants in the *RDHS* and *RPE65* genes), Oguchi disease (pathogenic variants in the *SAG* and *GRK1* genes), or mutations in the *GUCY2D* gene;
3. An **abnormal** fundus with changes typical of **axial myopia and nystagmus** suggests the following:
  - cCSNB—pathogenic variants in the *GRM6*, *TRPM1*, *LRIT3*, or *GPR179* genes are suspected;
  - icCSNB—pathogenic variants in the *CABP4*, *RIMS2*, or *CACNA1F* genes are suspected.
  **In X-linked inheritance:**
  - icCSNB—pathogenic variants in the *CACNA1F* gene—**nystagmus is common,** and fundus changes are possible:
    ○ Optic disc tilt, **optic nerve atrophy**, and the **morning glory syndrome** [12,13,14,15];
  - cCSNB—mutations in the NYX gene.
  **In autosomal dominant inheritance:**
  - **Normal** fundus and **no nystagmus**—pathogenic variants in the *GNAT1*, *PDE6B*, or *RHO* genes.

**Figure 1:**
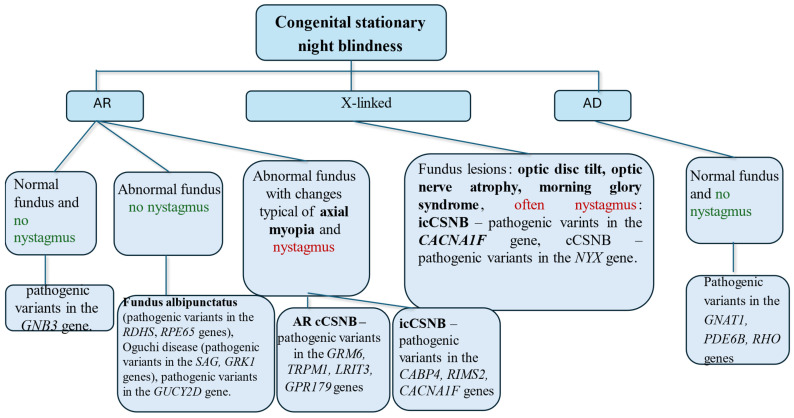
The diagnostic algorithm for CSNB, considering the inheritance type and the presence of nystagmus.

Data useful for diagnosing CSNB are presented in the diagram (Figure 1).

## 5. Case Reports and Review of Current Knowledge

### 5.1. Section 1 Cases Report

#### 5.1.1. Case 1

**Figure 2:**
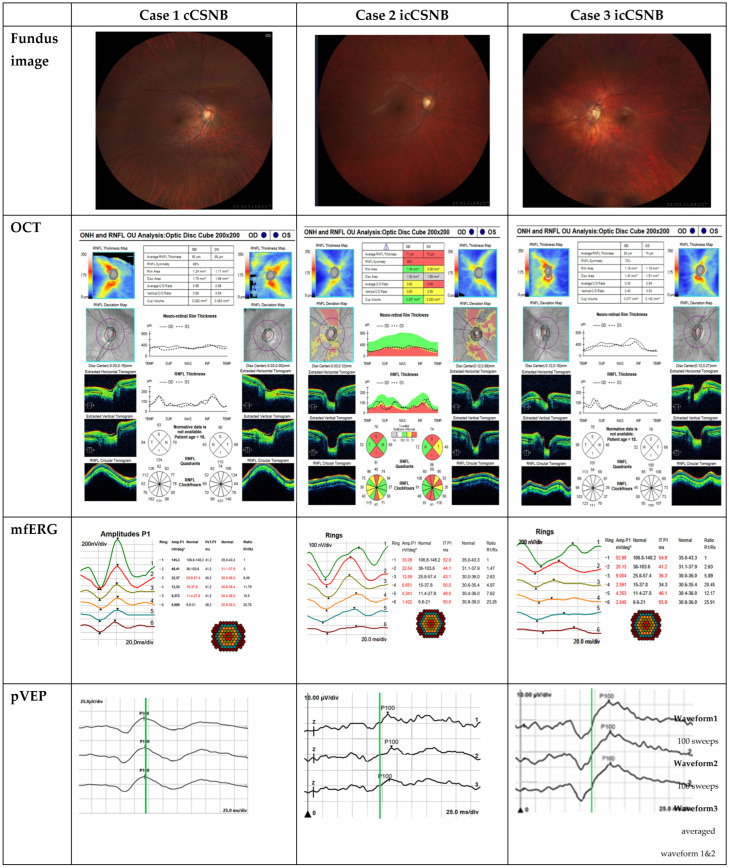
Photos showing the results of 3 cases with CSNB. Presented are fundus photos, OCT results of the optic discs, mfERG results (results marked in red are outside the normal limits; results marked in black are within the normal range), and pVEP results (the green line marks the upper limit of normal at 119.0 ms).

A 16-year-old female patient was referred to the ophthalmology clinic due to poor night vision since birth. The results of the examinations carried out are shown in Table 3, while photographs of the procedures performed are included in Figure 2.

In the available literature, OCT changes in cCSNB have not been described.

In electrophysiological tests (performed according to ISCEV standards), all features of cCSNB were present (Table 1), showing a defect in ON bipolar cells (Table 4). In the multifocal ERG (mfERG), cone system dysfunction was registered; 5′–30′ from the central point in rings R2–R6 a reduction in the P1 density response associated with an increase in the implicit time of the P1 wave was registered, most likely indicating dysfunction of the cone system with a defect in ON-type receptors.

The combination of pVEP results with mfERG (showing a normal response from R1) suggests a mild dysfunction or impaired signaling of ganglion cells or axons originating from the fovea.

Genetic testing was performed to confirm the diagnosis—next-generation sequencing (NGS) confirmed a variant in both alleles of the *GPR179* gene, the pathogenic variant NM_001004334.3:c.984delC (p.Ser329ProfsTerX). This pathogenic variant is associated with cCSNB, an autosomal recessively inherited disorder. Perhaps the characteristic feature of this mutation variant is partial atrophy of the optic nerve.

#### 5.1.2. Case 2

A 20-year-old male was referred to an ophthalmologist for refractive error control (myopia diagnosed in childhood), additionally reporting poor vision in the dark. The results of the examinations that were carried out are shown in Table 3, while photographs of the procedures performed are included in Figure 2.

Electrophysiological tests (performed according to ISCEV standards) showed all features of icCSNB (Table 5), revealing defects in both ON and OFF bipolar cells.

In the pVEP 0°16′ examination, prolonged P100 wave latency was observed—130.1 ms (high limit to 119.0 ms)—and in mfERG, abnormal results were also found in the first ring, as compared to cCSNB.

DNA analysis revealed a pathogenic variant in one allele (copy) of the *CACNA1F* gene, which has been associated with optic nerve atrophy in the literature [14]—according to the American Society of Human Genetics (ASHG) and the Human Genome Variation Society (HGVS). It is a pathogenic variant: NM_005183.4:c.2576+1G>A. Given that CACNA1F loss-of-function variants are well-documented causes of X-linked congenital stationary night blindness, this variant, according to American College of Medical Genetics (ACMG) Guidelines, is most likely Class 5 (Pathogenic).

#### 5.1.3. Case 3

The 12-year-old brother of the above-mentioned patient reports that he has “always been afraid of the dark”. He wears glasses due to myopia. The results of the examinations carried out are shown in Table 3, while photographs of the procedures performed are included in Figure 2.

In the OCT of the optic disc, we observed RNFL thinning of the RE and LE. Currently, there are no standards for OCT results for children, but based on the 2020 article “Normative data for optical coherence tomography in children: a systematic review” Banc A., Ungureanu M, the standards for age 12 (performed on ZEISS Optical Coherence Tomography which we also performed in this study) are about an average of 106.79  ±  12.98, and in our patient, they amounted to 74 in the LE and 80 in the RE.

Electrophysiological tests (performed according to ISCEV standards) showed all features of icCSNB (Table 6), revealing defects in both ON and OFF bipolar cells. mfERG RE/LE showed dysfunction of the cone system’s bioelectrical function across the entire analyzed area.

In the pVEP and mfERG, abnormal results were also found in the first ring, similar to his brother as mentioned above.

DNA analysis was performed, and a pathogenic variant was found in one allele (copy) of the *CACNA1F* gene, a variant associated with icCSNB, namely NM_005183.4: c2576+1G>A, the same pathogenic variant as in his brother, which is inherited in an X-linked manner. According to ACMG guidelines, it is most likely Class 5 (Pathogenic).

### 5.2. Section 2 Review of Current Knowledge

#### Conditions with Electronegative ERG

A negative ERG is also observed in other disease entities that need to be considered in the differentiation of CSNB.

The differential diagnosis of the negative type electroretinogram, divided into unilateral and bilateral occurrence, is presented in Table 7 [3,16].

## 6. Discussion

Establishing a diagnosis of CSNB based solely on a standard ophthalmological examination, especially in children without a positive family history, is often not possible [12]. The fundus in CSNB may be normal, but the ERG is abnormal in all patients and is crucial for establishing diagnosis.

The negative ERG is characteristic for all types of CSNB [8,11,17] and suggests impaired transmission between photoreceptors and bipolar cells [18]. In CSNB, the ERG and extended ON-OFF ERG help distinguish between the complete and incomplete forms.

In our article, we present a case of cCSNB characterized by a deficit in the ON-bipolar cell response, as well as patients with icCSNB with deficits in both the ON and OFF bipolar cell responses. The obtained results are consistent with those described in the literature [7,19,20].

An additional differentiating tool for these forms, which we did not apply but is described previously, is the S-cone ERG test—absent in cCSNB and present but abnormal in icCSNB [17]. These differences are due to the fact that, in cCSNB, the short-wavelength cone system is more affected than the long- and medium-wavelength cone systems [21].

### 6.1. Electrophysiological Studies in CSNB Available in the Literature

#### 6.1.1. Flicker ERG

Y Miyake et al. published the results of a study of ten patients with icCSNB examined using flicker ERG (30 Hz) [22]. In all patients, the ERG showed an excessive increase in amplitude with the occurrence of a characteristic splitting of the upper peak of the wave as light adaptation progressed. The mechanism of the increase in cone ERG amplitude during light adaptation is not fully understood. The flicker ERG results were normal in patients with cCSNB, which is consistent with the literature. In our cases of icCSNB, as reported in the literature, the ERG showed a splitting of the b-wave upper peak along with a reduction in interpeak values. The described excessive increase in interpeak values with prolonged adaptation time was not tested by us.

#### 6.1.2. MfERG

Multifocal ERG is generally used to diagnose macular dysfunction. It allows for the recording of electrical signals from multiple distinct areas of the retina, enabling topographical visualization of retinal function [23]. The mfERG provides information about the bioelectrical function of ON and OFF bipolar cells and, to a lesser extent, cones. Therefore, some researchers have also performed mfERG on patients with CSNB.

Mineo Kondo et al. in 2001 observed that patients with cCSNB are characterized by the following:

1. **Normal P1 wave amplitude with prolonged peak time in the foveal area (R1);**
2. **Significant reduction in P1 wave amplitude in the parafoveal area (R2)** [24].

It was observed that in cCSNB, the second kernel response is reduced, which was explained by abnormalities in the postsynaptic ON pathway, and this may cause prolonged peak times in the first kernel response. Similar mfERG results were also obtained by Francois Tremblay et al [18]. In our described case of cCSNB, we obtained the following first-order kernel results in the mfERG examination:

1. Normal amplitude with normal peak time in the first ring;
2. Normal amplitude with prolonged peak time in the second ring;
3. Reduction in amplitude in rings 3–5 and prolonged peak time in rings 2–6.

The differences between the results obtained in our study and those obtained by other researchers may be related to the different pathogenic variants described in the literature for cCSNB cases, which could influence the variability of bioelectrical disturbances in the mfERG. In the mfERG examination of patients with icCSNB, researchers observed that different mutations cause varying changes in the cone system. Responses from the fovea have a P1 component with normal amplitude and peak time, while those in more peripheral locations show a reduced amplitude and significantly delayed peak time [18]. The discrepancy between these mfERG responses suggests that cones in the fovea have access to compensatory mechanisms that partially restore their synaptic terminals to Ca^2+^ homeostasis mechanisms, which are not available to more peripheral cones.

In our two cases of patients with icCSNB and a documented mutation in the *CACNA1F* gene, variant NM_005183.4: c2576+1G>A, we demonstrated dysfunction in the cone system which manifests as an altered bioelectrical function and prolonged peak time across the entire analyzed area. The aforementioned authors did not describe this mutation variant, suggesting that different mutations in the *CACNA1F* gene may contribute to various signal transmission disorders between cones and bipolar cells. Consequently, this could lead to distinct bioelectrical dysfunctions, as observed in mfERG.

#### 6.1.3. VEP

In the pattern VEP (pVEP) response of patients with cCSNB, Claire S. Barnes et al. observed a prolonged p100 wave latency at reduced contrast levels of 10%, 20%, and 50% [25]. The authors of this study noted that their described case did not have an identified mutation.

We were unable to compare our pVEP results, which were obtained according to ISCEV standards at 97% contrast (ours were performed to the standard that ISCEV recommends, ≥80%) [26]. In our case of cCSNB, the VEP result was normal, indicating that using high-contrast stimulation is not useful for detecting defects in cCSNB, and that different mutations in the same condition may be responsible for varying signal transmission disorders. At the same time, the normal pVEP and mfERG results from the first ring show that the entire visual pathway after foveal stimulation in our patient with cCSNB and a mutation in the *GPR179* gene, namely variant ENST00000342292, is functioning normally.

In icCSNB with a *CACNA1F* pathogenic variant, consistent with Takahashi [27], we observed a prolonged p100 wave latency in pVEP. The observed changes in pVEP are likely due to cone system dysfunction, as evidenced by the mfERG results showing reduced R1 response density and prolonged peak time of the p1 wave.

There is one available publication on flash VEP stimulation in patients with icCSNB and a *CACNA1F* gene mutation, namely variant c.1218delC [28,29]. Researchers demonstrated that some patients exhibit persistent crossed asymmetry, typical of the abnormal optic nerve routing seen in albinism. The cause of this VEP asymmetry was not identified, and no pathology of the optic nerve was found that could explain it. Imaging studies—head and orbit MRI—showed no abnormalities.

#### 6.1.4. Electrooculography (EOG)

Takamashi, Mineo Kodo, et al. [24,27] consistently demonstrated normal EOG results and a normal Arden ratio in patients with cCSNB and icCSNB. Christina Zeitz et al. performed EOG in autosomal dominant cases of CSNB with mutations in the *RHO*, *PDE6B*, and *GNAT1* genes, obtaining results within the normal range, indicating normal retinal pigment epithelium function in this condition [30].

#### 6.1.5. Pattern ERG

Panagiotis et al. performed PERG on six patients with cCSNB and mutations in the GRM6 gene with different mutation variants. In one patient, the result was normal, in two it was subnormal, and in two it was absent. The obtained results are further evidence that both the gene mutation and the mutation variant affect the function of the gangiol cells and cone in the macular region [31].

### 6.2. Combined Structural and Functional Exams Improve Diagnostics of CSNB

Combining electrophysiological tests with multimodal retinal imaging can help make a more precise clinical diagnosis, even in cases where the fundus appears normal or nearly normal [12,16]. As in our described cases of icCSNB, performing OCT was one of the tests that partially confirmed optic nerve atrophy, and in cCSNB, it allowed for the detection of fiber loss corresponding to subtle changes in the clinical examination. Other researchers [32] have demonstrated a normal OCT result in a patient with cCSNB and a mutation in the *GPR179* gene (as in our case), but with a different variant (c.1811C>T leading to p.(P604L) change in exon 9), which is why we are unable to compare these results.

### 6.3. Genetics in CSNB

To date, 18 different genes (*GNAT1*, *PDE6B*, *RHO*, *SLC24A1*, *NYX*, *GRM6*, *TRPM1*, *GPR179*, *LRIT3*, *CACNA1F*, *CABP4*, *CACNA2D4*, *RDH5*, *RLBP1*, *RPE65*, *SAG*, *GNB3*, and *GRK1*) with over 360 different pathogenic variants and more than 670 defective alleles have been identified in association with CSNB, including genes encoding proteins of the phototransduction cascade [5,33]. With the advancement of genetic research over the past decade, the molecular diagnosis of CSNB has become more precise through the identification of relevant gene mutations. In the complete variant of CSNB (cCSNB), mutations in the *TRPM1*, *NYX*, *GRM6*, *LRIT3*, and *GPR179* genes have been identified, whereas mutations in the *CACNA1F* and *CABP4* genes are associated with the development of the incomplete variant of CSNB (icCSNB) [4]. Different phenotypic features may be associated with different defects of the same gene [12].

*CACNA1F* is a gene with 48 exons that encodes the α1 subunit of the voltage-dependent L-type Ca^2+^ channel—a large protein up to 1966 amino acids in length [5,11]. Voltage-dependent L-type Ca^2+^ channels are located in photoreceptors at the junction with bipolar cells. To date, Hoda and others, as well as Stockner and Koschak, have identified over 50 mutations in *CACNA1F* that may affect voltage-dependent L-type Ca^2+^ channels, altering either protein expression levels or channel function [34,35]. The founder variant of *CACNA1F*, c.3166dupC (c.3167_3168dupC), has been reported in individuals of Dutch–German–Mennonite origin [36]. The NM_005183.4 variant of the *CACNA1F* gene, with pathogenic variants c.3847-2A>G and c.3940C>T (p.Arg1314Cys), was first described in 2022 in a case of X-linked cone–rod dystrophy (CORDX), a rare, progressive retinal disease characterized by reduced visual acuity, myopia, abnormal color vision, and cone–rod dystrophy [13]. Furthermore, other studies suggest that individuals with incomplete CSNB related to the *CACNA1F* gene often exhibit nystagmus, photophobia, and/or impaired night vision, abnormal color vision, myopia, and generally a normal fundus, except for changes typical of high myopia (optic disc tilt, disc pallor, and stretching of the fundus). There are rare reports of **optic nerve atrophy** associated with *CACNA1F*-related retinopathy [12,14,15,37]. In our case, we also demonstrated an association of this gene with optic nerve fiber atrophy, with the identified NM_005183.4: c2576+1G>A pathogenic variant. The *CACNA1F* gene may rarely be associated with **morning glory syndrome** [38]. According to the literature, the *CACNA1F* mutation accounts for about 55% of cases of X-linked CSNB [8].

The *GPR179* gene encodes a seven-transmembrane G-protein-coupled receptor, so it is not yet clear whether *GPR179* functions solely as a regulator or also serves as a co-receptor (for example, with *GRM6*) [5]. Genes involved in the depolarization of ON bipolar cells include *TRPM1*, *NYX*, *GPR179*—detected in our described patient—and *LRIT3*, which, when damaged, similarly cause a negative ERG and are associated with high myopia [5]. Michelle Hendriks et al. demonstrated a strong association between the occurrence of myopia in cCSNB and hyperopia in icCSNB [39], though the mechanism behind these associations is not known. Other studies suggest that myopia in cCSNB is primarily axial and may be related to altered dopamine secretion and amacrine cell dysfunction [40].

### 6.4. Monitoring

Due to the non-progressive nature of the disease, patients with CSNB do not require frequent ophthalmological examinations. To date, it is known that the night vision disorder does not progress over time, but due to comorbid refractive disorders—such as myopia—we suggest that patients with CSNB have annual eye examinations in the first decade of life and less frequently thereafter. Family planning presents an ideal opportunity to evaluate genetic risks and explore options for prenatal genetic testing. Providing genetic counseling to young adults, alongside career guidance, is both timely and beneficial during this stage [3]. The longest documented follow-up of a patient with CSNB in the literature is 38 years, which confirms the non-progressive nature of the disease [41].

Currently, causal treatment for CSNB is not available. There are ongoing attempts at gene therapy in animal models [42].

### 6.5. Treatment of Conditions Associated with CSNB

Coexisting myopia or hyperopia should be corrected as in other patients with refractive errors. In cases of accompanying nystagmus, the patient may position their head in a specific gaze direction (the so-called ‘null point’) to suppress the frequency of the nystagmus—in some cases, carefully planned strabismus surgery can shift the gaze position for the null point to a better functional range [3].

## 7. Conclusions

In the diagnosis of diseases manifesting with night vision disturbances, congenital stationary night blindness should be considered. An accurate diagnosis is especially important for the patient, as unlike other diseases that need to be included in the differential diagnosis, CSNB is non-progressive. The key diagnostic test in CSNB is the ERG. Knowledge about the phenotypic changes associated with different gene mutations is still very limited, and only a genetic diagnosis can ensure a correct diagnosis and offer adequate counseling for the patient and family. Genetic testing remains necessary, but detailed ophthalmological examinations that assess the structure and function of the retina often help guide genetic testing by indicating a single gene or group of genes for analysis, thus reducing diagnostic costs.

## Figures and Tables

**Table 1:** Comparison of ERG findings in cCSNB and icCSNB.

	cCSNB	icCSNB
DA 0.01 ERG	None	Reduced b-wave amplitude (subnormal)
DA 3.0 ERG	Negative ERG type: b-wave amplitude is smaller than the a-wave amplitude	Negative ERG type Analogous to cCSNB
LA 30 Hz	Normal	Splitting of the upper peak; reduction in interpeak values
LA 3.0 ERG	Broadened a-wave, normal amplitude	Subnormal response (reduced b-wave amplitude)
ON-OFF ERG	Abnormal response from ON bipolar cells; normal response from OFF bipolar cells	Abnormal ON and OFF response

**Table 2:** Comparison of schematic electrophysiological recordings in cCSNB and icCSNB compared to normal recordings.

ERG	cCSNB	icCSNB	Normal
**DA 0.01**	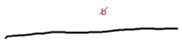	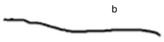	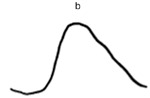
**DA 3.0**	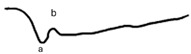	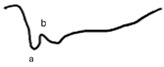	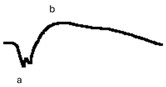
**LA 30 Hz**	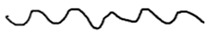	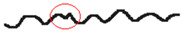	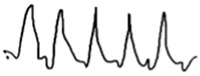
**LA 3.0**	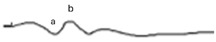	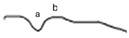	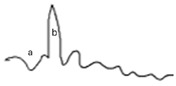
**ON-OFF** **response**	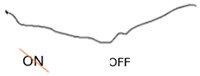	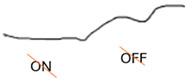	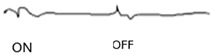

a—wave a, b—wave b.

**Table 3:** A table containing the most important information about the cCSNB and icCSNB cases. Other information is included in the text. Abbreviations: BCVA—best corrected visual acuity; RE—right eye, LE—left eye, OCT—optical coherence tomography, mfERG—multifocalERG, pVEP—pattern reversal VEP.

	Case 1 cCSNB	Case 2 icCSNB	Case 3 icCSNB
**BCVA RE [Snellen Chart]**	0.9	0.5	0.5
**BCVA LE [Snellen Chart]**	1.0	0.5	0.5
**Refractive error RE [D]**	−2.75	−3.5	−3.0
**Refractive error LE [D]**	−3.75	−2.5	−3.0
**Fundus examination**	changes indicative of axial myopia	optic disc cupping, slight temporal pallor in both eyes	optic disc pink, pale, temporally cupped, vertically oval, peripapillary pigment clumping
**OCT**	sectoral RNFL thinning	sectoral thinning in the superior and inferior regions; reduced GCC thickness in both eyes	thinning RNFL; reduced GCC thickness in both eyes
**Farnsworth Test**	normal	normal	normal
**Kinetic perimetry**	normal	normal	normal
**Intraocular pressure**	normal	normal	normal
**ON-OFF-response ERG**	defect in ON	defects in both ON and OFF	defects in both ON and OFF
**mfERG**	dysfunction 5′–30′ from central point R2-R6 reduction in P1 density response associated with an increase in the implicit time of the P1 wave	dysfunction of the cone system’s bioelectrical function across the entire analyzed area including the macular region	dysfunction of the cone system’s bioelectrical function across the entire analyzed area including the macular region
**pVEP**	normal	prolonged P100 wave latency	prolonged P100 wave latency
**Genetic testing**	*GPR179* gene, pathogenic variant ENST00000342292 homozygous	*CACNA1F* gene, NM_005183.4: c2576+1G>A, a pathogenic variant inherited in an X-linked manner	*CACNA1F* gene, NM_005183.4: c2576+1G>A, a pathogenic variant inherited in an X-linked manner

**Table 4:** Abnormal ERG recordings of RE of a patient with cCSNB (description in text) compared to normal recording. Z—stimulation onset, a—wave a, b—wave b. Note: two responses from stimulus condition (single white flash) are displayed to demonstrate the degree of consistency.

ERG	cCSNB	Normal
DA 0.01	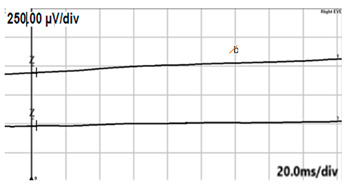	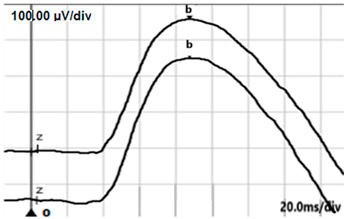
DA 3.0	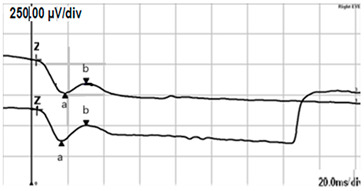	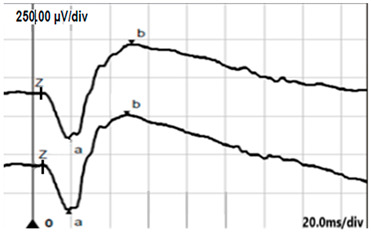
LA 30 Hz	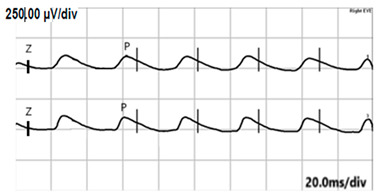	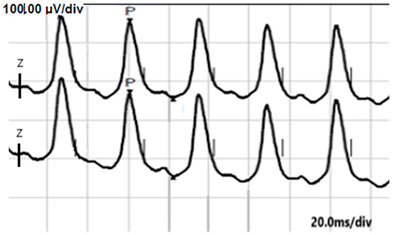
LA 3.0	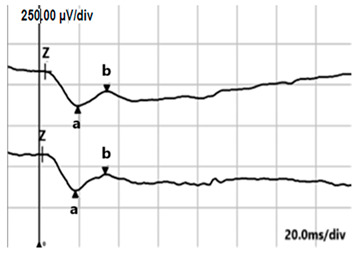	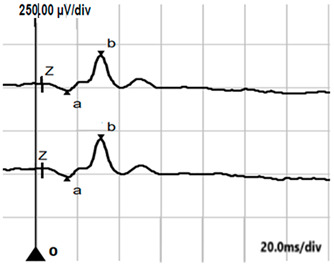
ON-OFF response	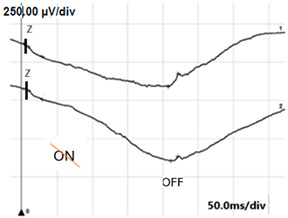	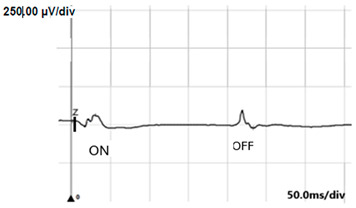

**Table 5:** Abnormal ERG recordings of the RE of a patient with cCSNB (description in text) compared to a normal recording. Z—stimulation onset, a—wave a, b—wave b. Note: two responses from the stimulus condition (single white flash) are displayed to demonstrate the degree of consistency.

ERG	icCSNB(1)	Normal
DA 0.01	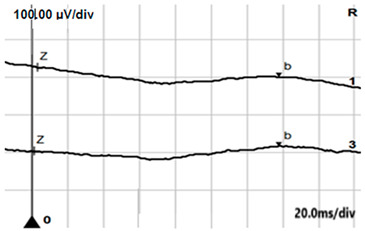	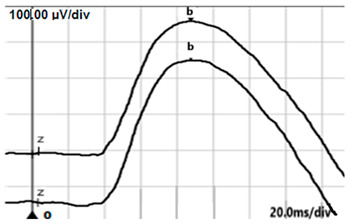
DA 3.0	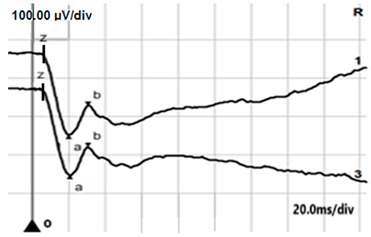	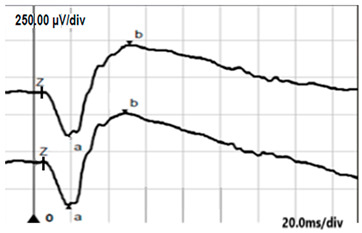
LA 30 Hz	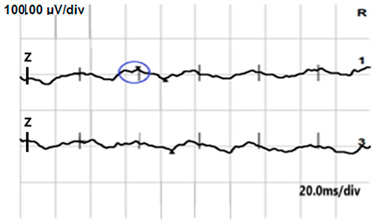	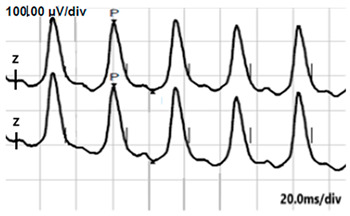
LA 3.0	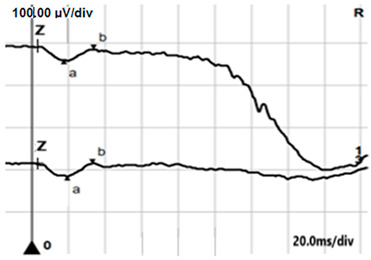	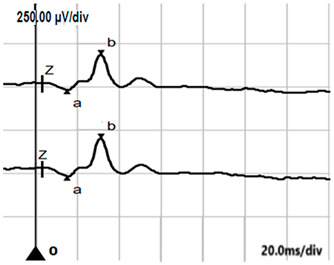
ON-OFF response	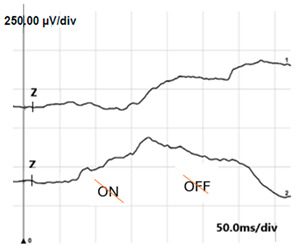	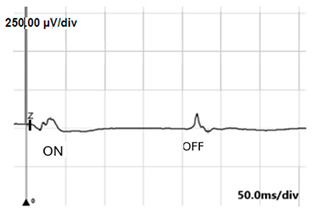

**Table 6:** Abnormal ERG recordings of the RE of a patient with cCSNB (description in text) compared to a normal recording. Z—stimulation onset, a—wave a, b—wave b. Note: two responses from the stimulus condition (single white flash) are displayed to demonstrate the degree of consistency.

ERG	icCSNB(2)	Normal
DA 0.01	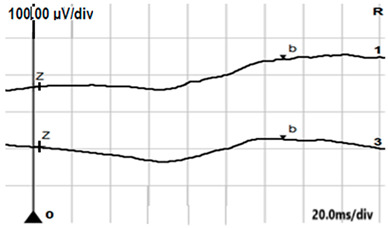	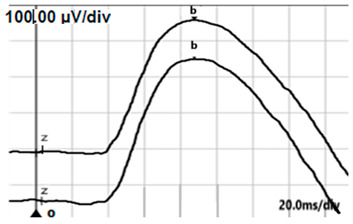
DA 3.0	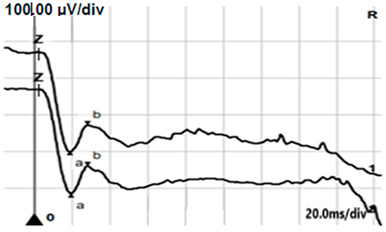	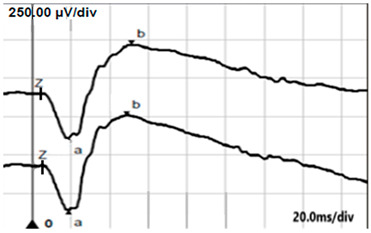
LA 30 Hz	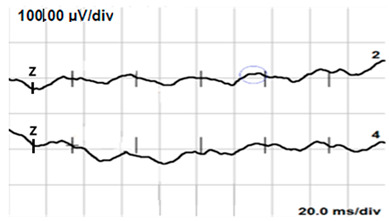	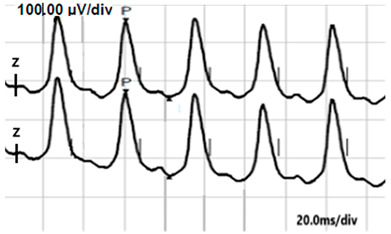
LA 3.0	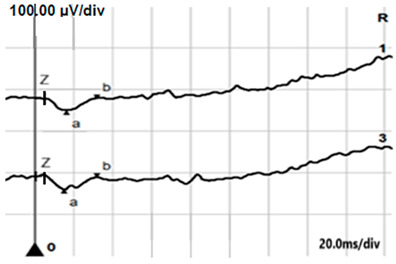	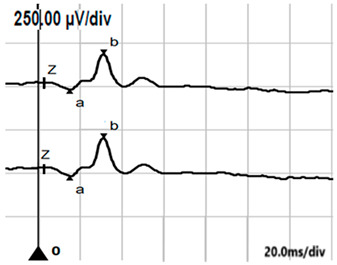
ON-OFF response	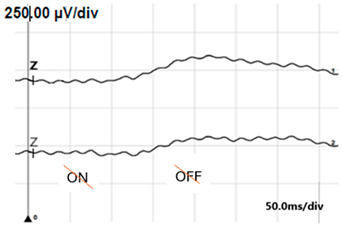	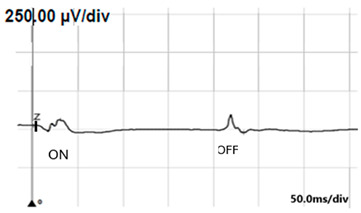

**Table 7:** Differential diagnosis of the negative type electroretinogram.

Unilateral	Unilateral/Bilateral Asymmetrically	Bilateral	
Ischemia (e.g., post-CRAO)	Autoimmune retinopathy (+MAR, CAR)	Vitamin A deficiency—acquired night blindness	
Siderosis	Birdshot chorioretinopathy	Photoreceptor dystrophies	
	Occlusive vasculitis	Retinoschisis	
		Batten disease (juvenile neuronal ceroid lipofuscinosis).	
		Vigabatrin	Methanol

## Data Availability

The data used to support the findings of this study are available from the corresponding author upon request.
